# Inquiry into the Physical Constitution of Water, the Structure of Fluids, and the Cause of Fluidity in General

**Published:** 1812-12

**Authors:** T. C. Speer

**Affiliations:** London


THE
### Medical and Physical Journal.

6 OF VOL. XXVIII.] DECEMBER, 1812. [NO. 166.

*To the Editors of the Medical and Physical Journal.*

GENTLEMEN,

FROM the great scope which your scientific publication affords for subjects of natural history and chemistry, as well as those which more immediately relate to medicine, I am induced to offer you the following Essay. It has been read before the Royal Physical Society of Edinburgh, and I have been induced to publish it at the earnest solicitation of some eminent and philosophical characters, whose judgment I highly value.

T. C. SPEER, M. D.

*London, October* 1812.

### *Inquiry into the Physical Constitution of Water, the Structure of Fluids, and the Cause of Fluidity in general.*

WHEN we fully and carefully estimate the extensive and important influence which water exerts in the economy of the animal, vegetable, and mineral, kingdoms, we must consider it as, perhaps, the most essential and universal agent which Nature has furnished for the support of her creation, and which man has obtained for the supply of his wants; hence, by many of the ancients, it was looked upon as the material principle of all natural bodies, and acquired the pompous title of *omni seminaria,* the universal seminary.

The most satisfactory way, probably, by which we can obtain this estimation, is by carefully investigating those simple laws of matter on which its nature, structure, and properties, depend; or, in other words, by inquiring into its *physical constitution.* This is the point of view under which I wish principally to consider this subject—its application in other ways, the various uses and effects of water in nature and the arts, and its history in the chemical world, are all well and fully known: any account of them here would be unnecessary.

The most distinguishing features in the character of water are, 1st, its facility of assuming the three natural states of bodies in general, viz. solid, fluid, and aeriform, according to the forces or intensities of that power by which its molecula are influenced; 2dly, that, as in each of these states its properties differ, these diversities are not proportioned to the variations of such power, but are separated from each other other by fixed and determinate limits: into each of these states, and their production, I shall now inquire.

Although the simple uncombined form of water is that of a solid, viz. ice, yet, in common language, our considerations of it are generally taken as a fluid. Under this view then, I shall begin my examinations of it; but, before doing so, I conceive it necessary to make some inquiry into the nature of fluids, and the cause of fluidity in general.

Boscovich's theory is, perhaps, the most rational that has ever been advanced on this subject. He thought that the atoms of fluids were placed in the limits between attraction and repulsion; that the distances between each atom were such that these opposite powers just balance each other; and that the form of these atoms was such that they can freely move without altering these distances. He supposes that the insensible distance between two atoms is made up of an indefinite number of alternate repulsions and attractions.

This hypothesis certainly seems highly ingenious, like all others adopted by its enlightened author; and, as a generalizing theory, seems perfectly satisfactory. However, it only refers to the state of a fluid ready formed; it does not inform us what are the progressive changes in fluidification; it does not tell us what are the specific transitions which occur in the states of the attracting and repelling powers when a solid body becomes a fluid body; and hence we do not know that standard by which the distances between atoms are regulated and varied.

In order to account more particularly for the phenomena of fluidity, and the changes it induces, we must take into calculation not only the change of *quantity*, but the change of *quality*, in those forces by which the atoms or particles are influenced. For a body to become fluid (from a state of solidity), it is not enough that its interstices should be rendered wider: the atoms themselves must assume a new form, arrangement, and position; new and different surfaces must be opposed to each other; the powers of cohering and repelling must be new modelled and changed in their line of direction, although this change is equal with regard to the whole mass, indeed this equality of action seems to be the *sine qua non* of fluidity.

In the conversion, therefore, of a solid into a fluid, it would seem that each particle is, by means of the repelling power, thrown by a sort of convulsive agitation (if I may be allowed the term) into such a position with regard to its neighbouring particles, that their force of cohesion acting on it equally on all sides, and as it were in straight lines or radii, radii, instead of taking one particular direction as they were accustomed to do, now approach to, and recede from, it at different and various angles of impingement, but with equal intensity as to the whole mass. The influence, therefore, of these cohering lines being equally diffused and directed around each atom, this atom must, of course, press equally in all directions upwards, downwards, sideways, &c.; in short, it may be said to form the centre of a circle, the line of circumference being formed by its neighbouring and surrounding atoms. A natural consequence, therefore, of this equality of action in the cohering forces all around each atom, must evidently be that the form of this atom is more or less globular. Indeed this would seem to be the last operation in the process of fluidification, and that hence the form of fluid particles depends on their cohesion, not the cohesion on the form.* In a solid, the pressure of each atom must be in one and the same direction, the angle of impingement one and the same, and hence the situation of each atom must be fixed and stationary; in a fluid, the pressure of each atom must be equal in all directions; and therefore each atom must have the power of motion.

Hence (as Dr. Black thought), to constitute fluid mobility in a body, it is necessary that the aggregation of its parts should be equal in every direction at the same distance, because in this case no force is necessary to move an adjacent particle from one situation to another, nor to keep the particle in its new situation with regard to the rest of the fluid.†

If fluidity merely depended on a change in the *quantity* of the cohering and repelling powers of the atoms, it should merely consist in an increase of interstice—a mere *expansion* or *increase of bulk*, and somewhat, perhaps, on the principle of a metal's expansion in the ratio of its ductility; the cohering lines being, as it were, put on the stretch, from the greater spaces they had to traverse. Now, we well know that expansion and fluidity are perfectly distinct; expansion is effected slowly and gradually; equal increments of it

* In tracing the course of operations performed so rapidly as that of fluidity, it is a nice and difficult matter to lay down the exact limit between cause and effect, between the antecedent and consequent, or to point out the exact line where the one ends and the other begins. The transition is so instantaneous, and, like the dependent links of one chain, they pass into each other with such rapid alternations, that they become almost unappreciable by the common measures of time or space.

† See Black's Lectures by Professor Robinson.

(generally-speaking) correspond with equal increments of temperature: fluidity is the sudden effect of a precise temperature; hence, therefore, the cohering powers must be changed not only in their quantity, but in their quality, or mode of action. Nay, if we go further, we shall find that this change of quantity and quality are by no means dependent on, or in a direct ratio with, each other; on the contrary, we know that some substances contract on becoming fluid, and *vice versa*,* as has of late been more fully and particularly illustrated by the ingenious experiments of Sir James Hall. † Another very remarkable effect attending this change of quality is, that, without it, the change of form in a body could not be influenced by any other power than those innate original ones with which all bodies are influenced, viz. attraction and repulsion; whereas, the moment that the quality or mode of action is changed, the body then becomes subject to the influence of other powers in the change of forms, and which powers are quite external, and foreign to it.

Of these powers, agitation is, perhaps, the principal. ‡ We know that, from the application of this power, very sudden changes in the production of solidity and fluidity arise; which changes must, I conceive, evidently arise from a correspondent suddenness of change in the direction of the cohering and repelling forces, and not from any change in the quantity or mass of these forces.

Hence, therefore, in the production of fluidity, I would attach more importance than what is generally assigned to this circumstance, viz. *change of quality*, or *change of action* in the cohering powers; although I am fully ready to allow that such change may primarily be superinduced by the energy of the original repelling power, which, of course, we suppose to be *caloric*. §

One

---

* Of this, water itself is a most remarkable instance, as will be seen hereafter.

† See the Edin. Philos. Transact. vol. vi.

‡ Mairan found, that, if we could cool water below 32° by agitation, it instantly froze.

§ Indeed, in our present state of knowledge, we cannot say what is the exact and particular cause, or combination of causes, which produces fluidity. We know too little of the nature and laws of corpuscular affinity. That some singular anomalies take place in these laws, is evident from the exceptions already alluded to of expansion by cold, and *vice versa*. This must prevent us from concluding that caloric is the sole primary cause of fluidity. We cannot say how

One of the grand distinctions between a solid and a fluid is, that the one has a gravitating centre, a spot on which, if freely balanced, it would remain at rest; the other has not: and in the former, viz. the solid, its parts are so connected as to form one and the same whole, one connected mass of integrancy; their united effort is concentrated, as it were, at one single point—*the centre of gravity*,—which, in a round body, is that part at which, if radii were drawn from the extremities, they would meet as at a focus; and, in a flat one, that part where lines drawn through the length and breadth would intersect each other; if a line drawn from this centre (or line of direction, as it is called) be within the base of the body, the body is balanced; if without, it falls, its equilibrium being lost. All solids, then, must have this central spot, because their particles are dependent on each other, they are fixed and immoveable. Their shape depends on the direction of their cohering lines; they present sides and surfaces, rough, as it were, for the insertion and approximation of other particles; the interstices are closely blocked up, and thus the collective force is concentred in one spot—the centre of gravity. When this body becomes fluid, circumstances are quite altered; the particles become wide asunder, the force of the cohering lines becomes equal in all directions; hence, the shape of the atoms becoming more or less globular, and the motion free, they become more and more independent of each other, each atom assumes to itself a centre of gravity; the gravitating lines proceed from the circumference to the centre, and this centre is to the line of circumference as the whole particle is to those around it. Thus then the whole mass of a fluid presents a series of gravitating foci, which in the solid had been collected and condensed into one; and thus a fluid may be said to consist of *a series of circles or globes rolling through each other.*

The pressure of fluids must therefore be not only perpendicular like solids, but upwards, sideways, and in all directions; their surface must always find its own level, and this level must be always parallel with the horizon.

The above remarks on fluidity I conceived necessary, because water is generally met with in this state, and because they more particularly refer to those simple laws of material structure on which its constitution depends. One of the

---

how far other powers are concerned; it is not impossible, nor indeed improbable, as Dr. Irvine thinks, but that electricity may have some influence: we know that water is a very good, ice a very bad, conductor of it.

principal principal agents in the support of these laws is cohesion; this can only subsist between homogeneal atoms; in no body are the atoms more homogeneal than in water, and hence water affords the most pure and perfect result of the laws of their material structure.'

(To be continued.)

